# The need for a complete dental autopsy of unidentified edentulous human remains

**DOI:** 10.1080/20961790.2021.1962038

**Published:** 2021-09-27

**Authors:** Emilio Nuzzolese, Mario Torreggianti

**Affiliations:** a Department of Public Health Sciences and Pediatrics, University of Turin, Turin, Italy; b Private Dental Clinic, Teramo, Italy

**Keywords:** Forensic sciences, forensic odontology, identification, dental autopsy, edentulous, missing person

## Abstract

In December 2017, a decomposed unidentified body was found near the river Tronto in Teramo, Italy. The corpse was found without any identifying documents or specific personal belongings, except for a packet of cigarettes. The medical examiner determined a gastric perforation from the intake of hydrochloric acid to be the cause of death. A jar of muriatic acid found near the body led to suicide being considered the manner of death. The Penal Court in Teramo appointed two forensic odontologists to complete the postmortem assessment and collect dental data for personal identification. The corpse was found wearing a complete set of upper and lower dentures. The dental autopsy and 42 periapical X-ray images helped generate a biological profile of a man totally edentulous with upper and lower dentures, as well as an osteosynthesis with two plates and screws in the left ascending ramus of the mandible. In March 2018, the sister of a missing person reported the disappearance of her brother, and a presumptive identification was performed through visual recognition of the decomposed body. The sister confirmed the presence of two dentures and the location of the maxillo-facial surgery for the treatment of the fractured mandible. A complete dental autopsy was able to establish his identity without any DNA comparison needed. This case highlights the importance of performing a complete dental autopsy inclusive of dental radiographs, and its value in the identification of all unknown human remains even when totally edentulous.

A complete dental autopsy should be performed in all cases of human identification.

**Keypoints**
- Despite a corpse being edentulous, a complete dental autopsy can still be useful.
- Dental radiographs, such as bitewings, periapical images, panoramic radiographs, and CT scans, are recommended in all identification autopsies.

## Introduction

The scientific literature in the human identification field of forensic science highlights the importance of a multidisciplinary approach when performing this process, through the collection of primary identifiers (fingerprints, DNA, and dental data) and secondary identifiers [1]. The identification of unknown human remains may rely on circumstantial evidence and visual recognition, although best standards and International Criminal Police Organization (INTERPOL) recommendations should be applied in both single and multiple casualty scenarios. In some jurisdictions, postmortem dental collection is limited to dental examination without a dental autopsy performed by one or more forensic odontologists, nor X-ray imaging of the jaws [2, 3]. This could be the case with edentulous individuals for whom the medical examiner feels that involving a forensic odontologist is not valuable. Exceptions include cases with the presence of teeth, dental roots, dental treatments, or empty alveoli with missing teeth that were lost postmortem.

Despite these caveats, here we report a successful identification of an edentulous unidentified body. In this case, a satisfactory complete dental autopsy narrowed the search of reported missing persons in that specific geographical area and then helped establish the body’s identity.

### Case history

In Abruzzo, one of the 20 regions of Italy with over 1.3 million inhabitants, there are currently 3 961 reported missing persons [4] and seven sets of unidentified human remains, as highlighted by the December 2020 official report of the Italian Ministry of the Interior (National Italian database of unidentified human remains: https://rncni.clio.it).

In December 2017, a decomposed unidentified body was found near the river Tronto in Teramo, Italy. The corpse was found without any identifying documents or specific personal belongings, except for a packet of cigarettes. The medical examiner determined the cause of death to be a gastric perforation following the ingestion of hydrochloric acid. A jar of muriatic acid was found near the body, which led the manner of death to be considered suicide. After the judicial autopsy performed by the medical examiner, the Penal Court in Teramo decided to appoint two forensic odontologists to collect the postmortem dental data for personal identification. The deceased individual was found wearing a complete set of upper and lower dentures.

In March 2018, 3 months after the body was found, a woman reported that her brother, who lived alone, had gone missing. A presumptive identification was performed through a visual recognition of the partially decomposed corpse. The woman confirmed that her brother was wearing dentures and provided authorities with the name of the dentist. She also revealed that he had previous face trauma, as well as maxillo-facial surgery to treat the fractured mandible. The odontological assessment resulted in a preliminary generic biological profile, which then helped establish the identity of the corpse through comparisons of the radiological postmortem data with the antemortem panoramic X-ray and dental data received from the dentist and hospital. No DNA comparison was required in this case.

## Dental autopsy and odontological findings

**Figure 1.**
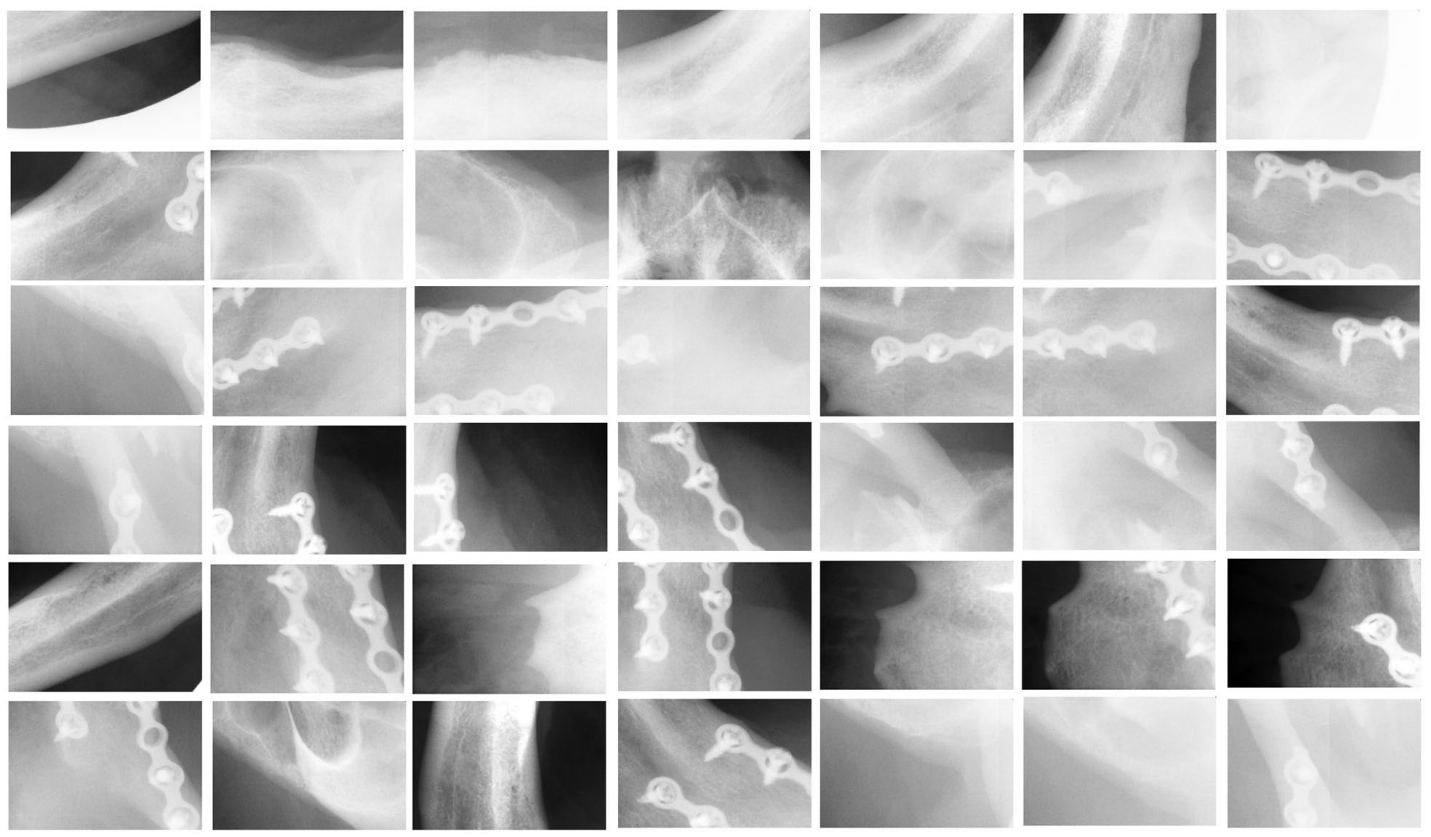
Forty-two periapical X-ray images obtained using a portable X-ray device coupled with a digital radiological sensor.

A dental autopsy was performed, which included an intra-oral examination with photographic images, denture images, and full dental radiography of the jaws. A portable X-ray device coupled with a digital sensor was used, and a total of 42 periapical X-Ray images were recorded (Figure 1). Dental findings helped generate a simple biological biography of a completely edentulous male with both upper and lower dentures, as well as an osteosynthesis with two plates and screws in the left ascending ramus of the mandible. The two dentures displayed mild wear of the resin teeth. Remarkable resorption of the alveolar crest, both superiorly and inferiorly, suggested that the man was wearing dentures for several years [5].

This profile was circulated by the Carabinieri Police Agency, then also sent to the regional association of missing persons, called “Penelope Abruzzo”, and to the editorial board of a national TV programme on missing persons, named “Chi l’ha Visto?” (“Have you seen this person?”).

## Radiological findings and definitive identification

**Figure 2.**
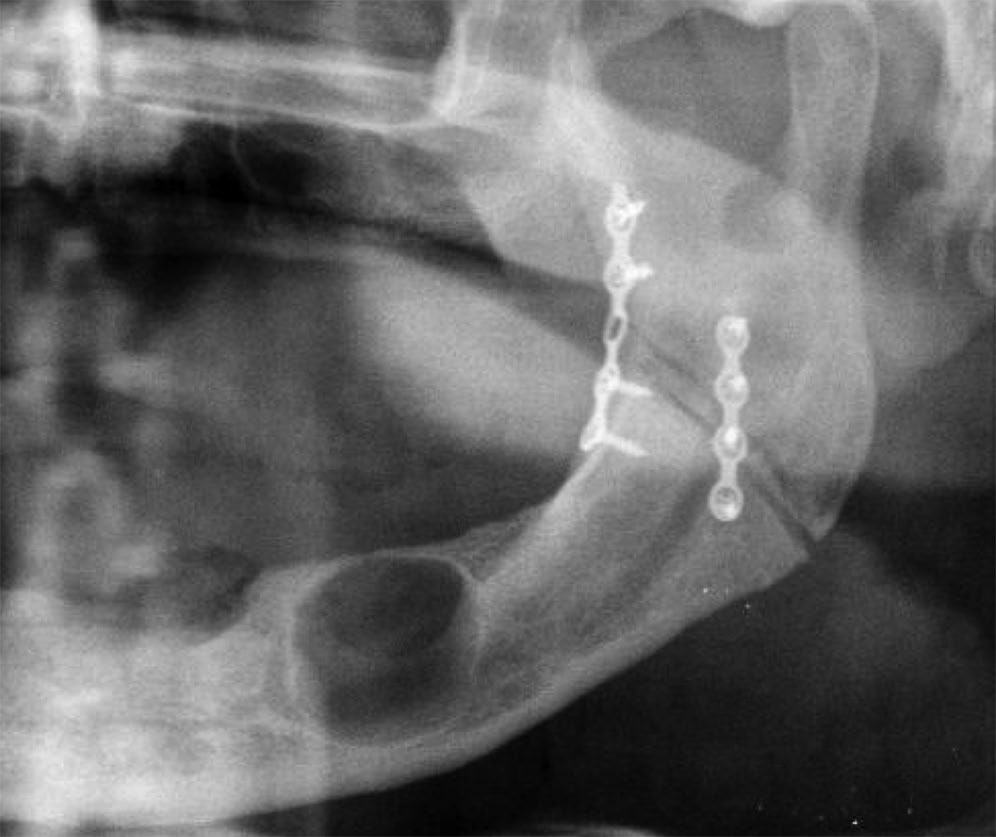
A close-up of the panoramic X-ray image of the patient received from the hospital, with the osteosynthesis in place on the left angle of the mandible.

**Figure 3.**
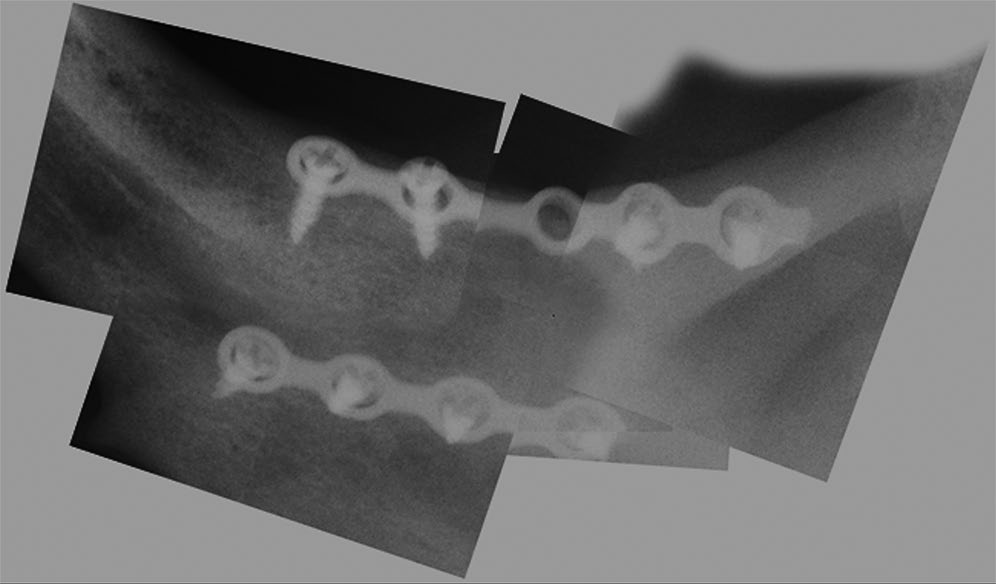
The collage of the periapical radiographs showing the osteosynthesis on the left angle of the mandible of the unidentified human remains.

The initial search for compatible reported missing persons did not offer any positive feedback, until the woman mentioned above reported her brother as missing after not hearing from him for several months. This was approximately 3 months after the body was found. An initial visual recognition of the partially decomposed corpse was performed with consideration given to sex and age compatibility. Further antemortem medical and dental data obtained from the sister revealed the name of her brother’s dentist, that he was wearing both upper and lower dentures, and that he had been hospita­lized for face trauma that resulted in a fractured mandible. By knowing the name of the hospital he was a patient in, the police was able to obtain his medical records. The records revealed that he was diagnosed with a complete fracture of the left angle of the mandible, which was treated by the maxillo-facial surgery unit. An antemortem radiograph highlighted an osteosynthesis of two plates and screws on the left angle of the mandible and a wide cystic pathology (Figure 2). Following the receipt of antemortem dental data, the two odonto­logists reassembled selected periapical X-ray images using a photo-editing software and obtained a wider radiologic view of the left angle of the mandible of the unidentified human remains (Figure 3). The comparison gave sufficient information to confirm a match between this individual and the remains. The man’s dentist was able to find the cast of his upper jaw, which was retained by his laboratory. The cast matched the upper denture of the unidentified human remains, specifically for shape and dimension of the second resin molar.

The compatibility of the preliminary biological profile and the comparisons with the antemortem and postmortem radiological dental data helped establish the identity of the corpse without having to collect a DNA sample.

## Discussion and conclusion

The requirement to perform a dental autopsy in each case of human identification where teeth and jaws are present incorporates best standards in human identification [6] and INTERPOL recommendations [7]. Forensic odontology plays a fundamental role in mass casualty situations [8]. The collection of postmortem dental radiographs, such as bitewings, periapical images, panoramic radiographs, and CT scans, is recommended not only in disaster victim identification [9] but also in single identification autopsies [10]. The goal of the human identification process is to compare and match antemortem data with postmortem findings. Although odontologists can establish a generic biological profile of an unidentified individual mostly by observing and analysing teeth and dental materials found in the jaws, many other relevant and identifying features can be retrieved through jaw examinations. Among the morphological and pathological identifiers of the jawbone [11], fractured teeth roots, retained endodontic material, or amalgam tattoos could also be present [12, 13]. Therefore, a prudent approach is necessary when comparing heterogeneous radiographic images [14] to achieve a positive identification with primary and secondary identifier pattern matches [7, 15–17]. In cases where no teeth are clinically visible during the dental examination, information gathered from dentures [18, 19] and other dental findings may assist in the identification process. Even when dentures were worn but not found in the unidentified human remains, changes in the oral mucosa consistent with the presence of dentures can be observed [20]. Despite the condition of edentulous jaws, this case highlights the importance of performing a complete dental autopsy inclusive of dental radiographs and its value in the human identification process [12].

## References

[1] INTERPOL Guide 1. Disaster Victim Identification (DVI). INTERPOL [accessed 2021 Jan 15]. Available from: https://www.interpol.int/How-we-work/Forensics/Disaster-Victim-Identification-DVI

[2] Nuzzolese E. Dental autopsy for the identification of missing persons. J Forensic Dent Sci. 2018;10:50–54. 30122870 10.4103/jfo.jfds_33_17 PMC6080158

[3] Gruber J, Kameyama MM. [Role of radiology in forensic dentistry]. Pesqui Odontol Bras. 2001;15:263–268. Portuguese. 11705275 10.1590/s1517-74912001000300014

[4] Italian Ministry of Interior. Special Commissioner for Missing Persons, 24th annual report (1 January 2020–31 December 2020) [accessed 2021 Feb 21]. Available from: https://www.interno.gov.it/sites/default/files/2021-02/xxiv_relazione_annuale_2020_compressed.pdf. Italian.

[5] Alsaggaf A, Fenlon MR. A case control study to investigate the effects of denture wear on residual alveolar ridge resorption in edentulous patients. J Dent. 2020;98:103373. 32389732 10.1016/j.jdent.2020.103373

[6] Pretty IA, Sweet D. A look at forensic dentistry—part 1: the role of teeth in the determination of human identity. Br Dent J. 2001;190:359–366. 11338039 10.1038/sj.bdj.4800972

[7] INTERPOL. Annexure 12: methods of identification [accessed 2021 Feb 21]. Available from: http://www.interpol.int/en/content/download/5759/file/E_DVIGuide2018_Annexure12.pdf

[8] Hill AJ, Hewson I, Lain R. The role of the forensic odontologist in disaster victim identification: lessons for management. Forensic Sci Int. 2011;205:44–47. 20950970 10.1016/j.forsciint.2010.08.013

[9] Viner MD, Robson J. Post-mortem forensic dental radiography—a review of current techniques and future developments. J Forensic Radiol Imag. 2017;8:22–37.

[10] Nuzzolese E, Lupariello F, Ricci P. Human identification and human rights through humanitarian forensic odontology. Int J Forensic Odontology. 2020;5:38–42.

[11] Du H, Li M, Li G, et al. Specific oral and maxillofacial identifiers in panoramic radiographs used for human identification. J Forensic Sci. 2021. 66:910–918. 10.1111/1556-4029.14673 33506528

[12] Slabbert H, Ackermann GL, Altini M. Amalgam tattoo as a means for person identification. J Forensic Odontostomatol. 1991;9:17–23. 1814936

[13] Berketa JW, Sims C, Al Adawiyah Binti Rahmat R. The utilization of small amounts of residual endo­dontic material for dental identification. J Forensic Odontostomatol. 2019;37:63–65. 31187744 PMC6875245

[14] Chiam SL, Page M, Higgins D, et al. Validity of forensic odontology identification by comparison of conventional dental radiographs: a scoping review. Sci Justice. 2019;59:93–101. 30654974 10.1016/j.scijus.2018.08.008

[15] Marlin DC, Clark MA, Standish SM. Identification of human remains by comparison of frontal sinus radiographs: a series of four cases. J Forensic Sci. 1991;36:1765–1772. 1770345

[16] Monsour PA, Young WG. Variability of the styloid process and stylohyoid ligament in panoramic radiographs. Oral Surg Oral Med Oral Pathol. 1986;61:522–526. 3086788 10.1016/0030-4220(86)90399-3

[17] Simpson EK, James RA, Eitzen DA, et al. Role of orthopedic implants and bone morphology in the identification of human remains. J Forensic Sci. 2007;52:442–448. 17316248 10.1111/j.1556-4029.2006.00370.x

[18] Lundberg E, Mihajlovic NS, Sjöström M, et al. The use of panoramic images for identification of edentulous persons. J Forensic Odontostomatol. 2019;37:18–24. 31589592 PMC6981351

[19] Richmond R, Pretty IA. Antemortem records of forensic significance among edentulous individuals. J Forensic Sci. 2007;52:423–427. 17316244 10.1111/j.1556-4029.2006.00378.x

[20] Junning C, Rohana A, Li W, et al. Biomechanics of oral mucosa. J R Soc Interface. 2015;12:20150325. 26224566 10.1098/rsif.2015.0325 PMC4535403

